# Unusual Case of Simultaneous Vaginal and Rectal Prolapse in a Jennet: Clinical Presentation and Management

**DOI:** 10.1155/crve/1125413

**Published:** 2026-05-08

**Authors:** S. Saraiva, F. Silva, J. García-Díez

**Affiliations:** ^1^ Animal and Veterinary Research Centre (CECAV), School of Agricultural and Veterinary Sciences, University of Trás-os-Montes and Alto Douro (UTAD), Vila Real, Portugal, utad.pt; ^2^ Associate Laboratory for Animal and Veterinary Sciences (AL4AnimalS), School of Agricultural and Veterinary Sciences, University of Trás-os-Montes and Alto Douro (UTAD), Vila Real, Portugal, utad.pt; ^3^ Department of Veterinary Sciences, School of Agricultural and Veterinary Sciences, University of Trás-os-Montes and Alto Douro (UTAD), Vila Real, Portugal, utad.pt

**Keywords:** jennet, recurrence, rectal prolapse, vaginal prolapse

## Abstract

A 5‐year‐old adult jennet in late pregnancy was presented for evaluation of a mass protruding from the vagina that had been noticed approximately 8 h earlier. Physical examination revealed a large (15 × 14 × 12.5 cm), pink‐to‐red vaginal protrusion. The jennet was estimated to be approximately 2–3 weeks from parturition, and the remainder of the physical examination was unremarkable. A diagnosis of vaginal prolapse was made. The prolapse was manually reduced. Foaling occurred 15 days later, with veterinary assistance. Parturition was dystocic, characterized by bilateral forelimb flexion, and required obstetrical intervention. Two months postpartum, the jennet presented with recurrent vaginal prolapse and 1 month later, she subsequently developed a rectal prolapse concurrently with vaginal prolapse. In total, three prolapse events were observed (three vaginal and one rectal). Following parturition, the jennet failed to return to estrus. To the best of the authors′ knowledge, no previous cases of vaginal and rectal prolapse in a jennet have been reported.

## 1. Introduction

Vaginal prolapse has been described in the veterinary literature as a spontaneous protrusion of vaginal tissue during late pregnancy, typically presenting as a soft, pink mass protruding from the vulva [1]. Rectal prolapse is defined as the protrusion of one or more layers of the rectum through the anus [2]. These conditions are frequently encountered in livestock, particularly rectal and vaginal prolapse in pigs [3], and are also well documented in cattle and small ruminants [2]. In equids, although rectal prolapse is considered rare, it has been reported more frequently in donkeys than in horses [4]. The available literature on rectal prolapse in donkeys suggests that parasitic infection is the predominant underlying cause [5]. Vaginal prolapse accompanied by rectal prolapse during advanced pregnancy and in the postpartum period has been reported in cows [6]. Additionally, a single case report of concurrent vagino‐cervical and rectal prolapse has been described in a doe [2].

## 2. Case Presentation

**Figure 1:**
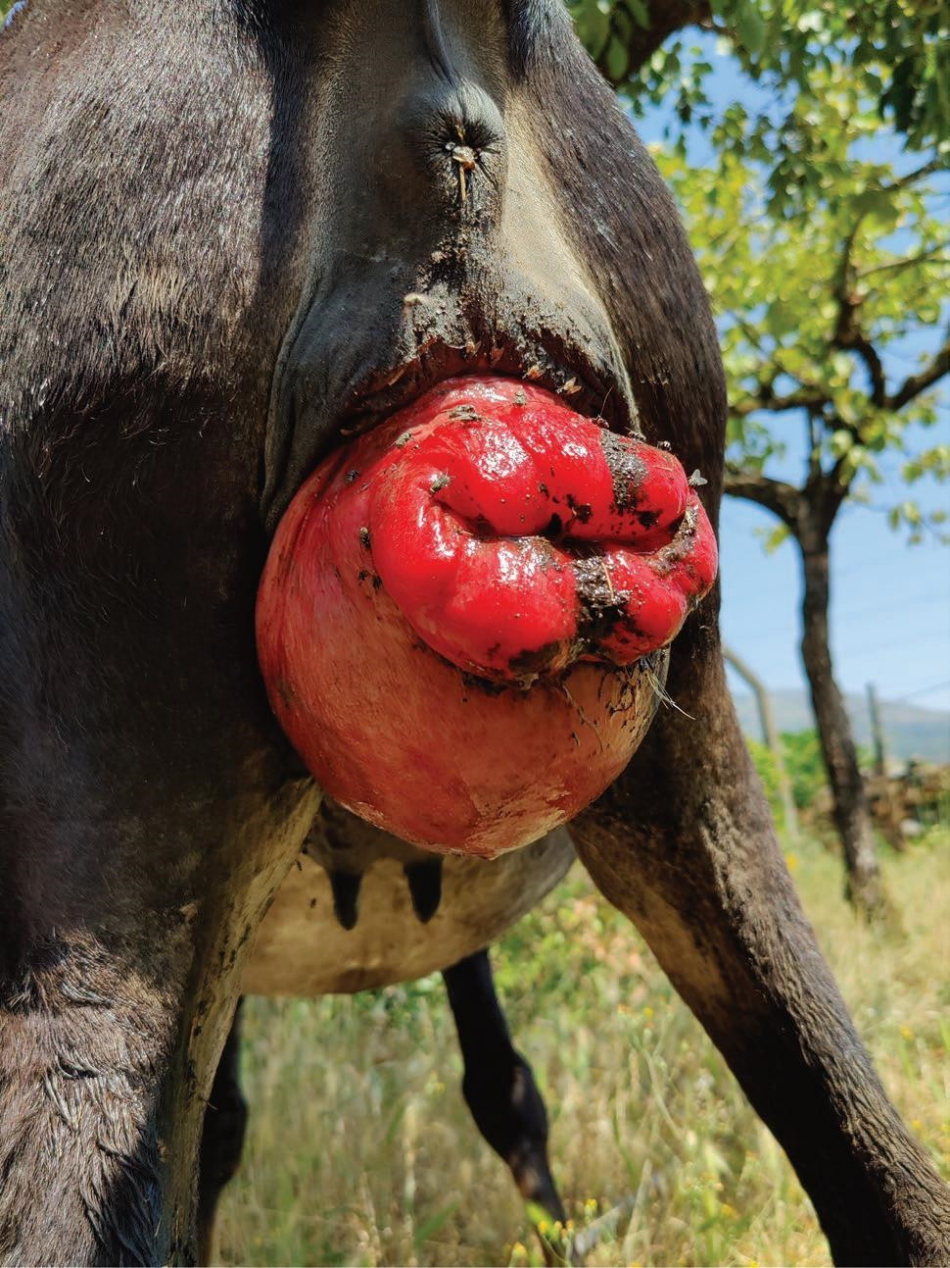
Vaginal prolapse.

A 5‐year‐old adult crossbred jennet (200‐kg body weight) in late gestation was presented for evaluation of a mass protruding from the vagina, first noted by the owner approximately 8 h before veterinary examination. Physical examination revealed a large (15 × 14 × 12.5 cm), soft, pink‐to‐red vaginal protrusion (Figure 1). A diagnosis of vaginal prolapse was established. The jennet was estimated to be approximately 2–3 weeks from parturition, and no additional abnormalities were identified on general physical examination. The prolapse was manually reduced under sedation, and a temporary vulvar retention suture was placed. The owner was advised to closely monitor the jennet for signs of impending parturition and to seek immediate veterinary attention if any occurred. The vulvar retention sutures were removed 7 days later without complications. Foaling occurred 10 days after suture removal and required veterinary intervention due to dystocia characterized by bilateral forelimb flexion. Two months postpartum, the jennet developed a recurrent vaginal prolapse, which was subsequently followed by rectal prolapse despite conservative management. At that time, fecal consistency and defecation frequency appeared normal, and no diarrhea or gastrointestinal disturbances were observed. The jennet also failed to return to estrus. The animal was appropriately vaccinated, including immunization against equine influenza and tetanus, and was regularly dewormed with oral ivermectin–praziquantel paste administered twice yearly.

### 2.1. Treatment

**Figure 2:**
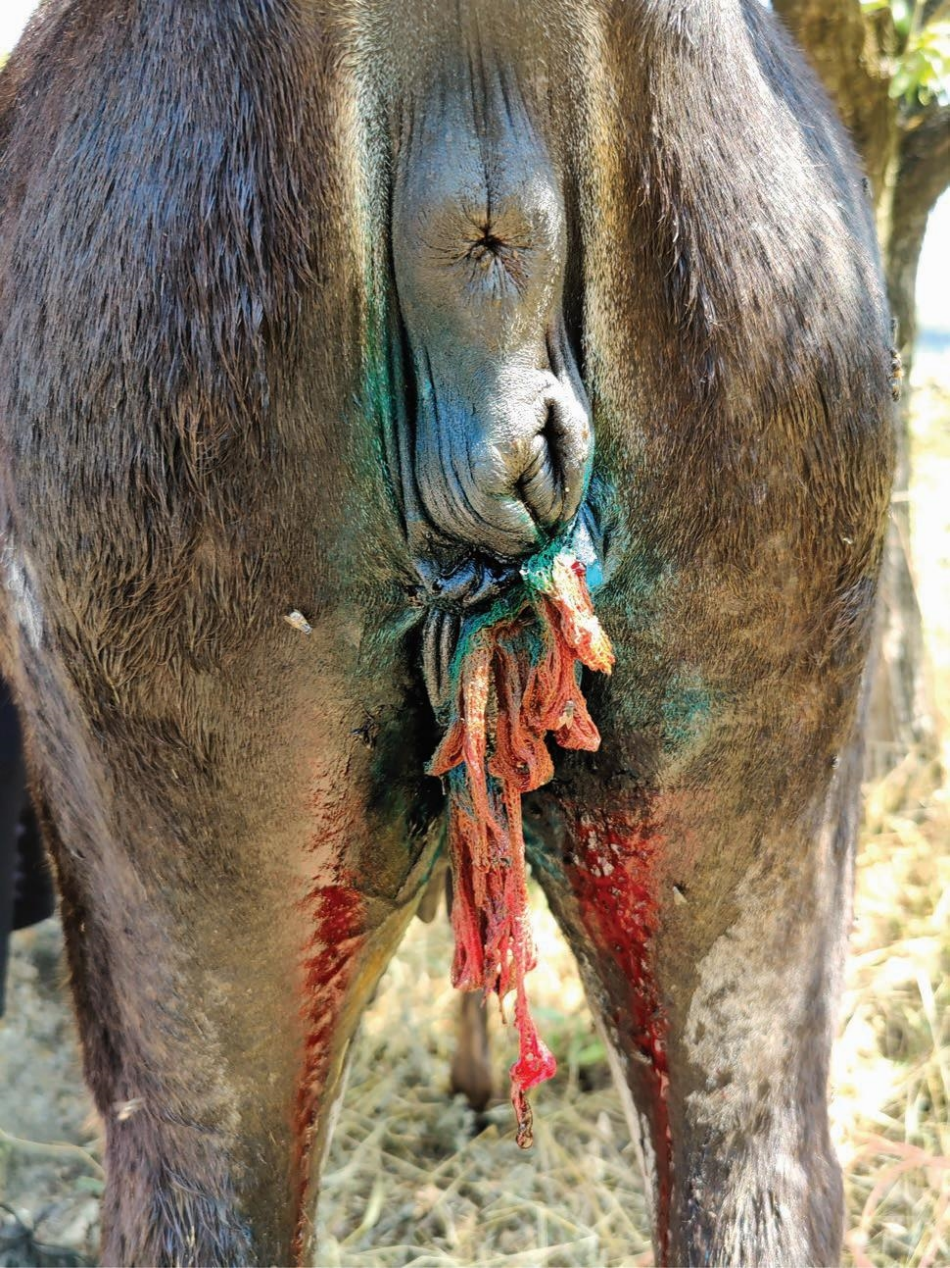
Prolapse reduction.

The vaginal prolapse was first cleaned and disinfected using a povidone–iodine vaginal solution (Betadine). Manual reduction of the prolapse was performed under sedation with xylazine (Xylexx, 0.05 mg/kg, i.v.). The labial tissue was desensitized with topical procaine hydrochloride (40 mg/m) combined with adrenaline tartrate (0.036 mg/mL) (Procamidor Duo). A temporary vulvar retention suture was placed to appose the labial edges (Figure 2), minimizing the risk of recurrence while maintaining adequate space for urination. Pain and inflammation were controlled with flunixin meglumine (Meganyl, 1.1 mg/kg, i.v., q24h for 3 days). Suture cleaning and disinfection was performed using chlorhexidine, followed by twice‐daily topical application of oxytetracycline spray (Terramicina) until suture removal. Stall rest and close monitoring were advised, with particular attention to clinical signs of parturition. The jennet was scheduled for a 7‐day re‐examination and suture removal. At that time vaginal tissue appeared normal, with no evidence of prolapse. The sutures were intact and a labial tear was repaired by surgical reconstruction. Foaling occurred 10 days after suture removal with veterinary assistance. Parturition was dystocic, characterized by bilateral forelimb flexion, and required active obstetrical intervention to ensure safe delivery. Following delivery, oxytocin was administered to facilitate assist in the expulsion of retained placental membranes, along with antibiotics and anti‐inflammatory medication. To prevent secondary infection, systemic antimicrobial therapy was instituted using a combined formulation of benzylpenicillin 12.000 IU/kg and dihydrostreptomycin 15 mg/kg (Pendistrep, 12mL, q24h for 5 days). The foal was in good condition and showed normal suckling behavior.

### 2.2. Outcome

**Figure 3:**
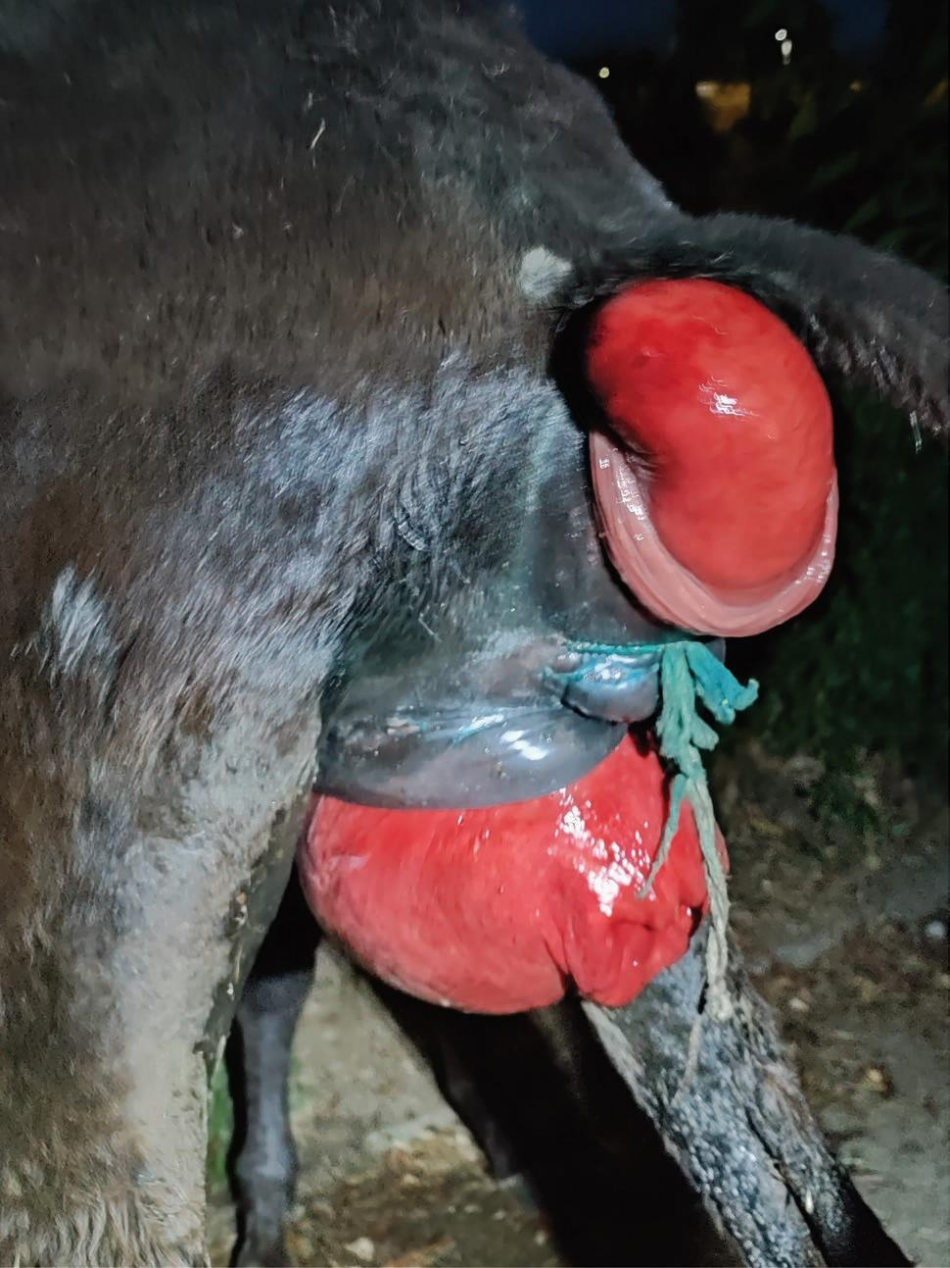
Prolapse vaginal and rectal Type III.

Two months after parturition, the jennet presented with a recurrent vaginal prolapse. Resolution was accomplished as previously described. Then, 1 month later, a recurrent vaginal prolapse associated with rectal prolapse was observed (Figure 3). Caudal epidural (Procamidor Duo) anesthesia to reduce tenesmus was applied (5mL, procaine chloride 40 mg/mL + adrenaline tartrate 0.036 mg/mL). The prolapsed rectal tissue was cleaned with warm isotonic saline and gently manually reduced to its normal anatomical position. No temporary purse‐string suture around the anus was applied. Vaginal prolapse was reduced as previously described. In addition, the jennet failed to return to estrus following parturition.

## 3. Discussion

In the present case report, the jennet presented with recurrent vaginal prolapse both before and after parturition. Pregnancy represents a significant risk factor for vaginal prolapse, which most commonly occurs during the final third of gestation due to increased intra‐abdominal pressure [7]. Hormonal changes, particularly increased estrogen levels, may also contribute to relaxation of pelvic and perineal tissues and predispose to vaginal prolapse [1]. Additionally, advanced age appears to increase susceptibility to prolapse, possibly due to reduced elasticity of connective tissues and weakening of pelvic support structures [7]. In the present case, vaginal tissue laxity combined with increased intra‐abdominal pressure may have contributed to the initial prolapse. Jennies that experience uterine or vaginal prolapse may be predisposed to recurrence in subsequent parturitions because each pregnancy reactivates relaxin‐mediated softening of pelvic support tissues, including pelvic ligaments and associated connective structures [8]. However, recurrence rates in donkeys remain poorly documented due to the rarity of the condition, and in some cases, the underlying cause cannot be clearly identified [7].

In this report, recurrent vaginal prolapse and a Type III rectal prolapse were observed 3 months postpartum, suggesting progressive compromise of the perineal and pelvic support structures. Type III rectal prolapse was characterized by prolapse of the rectal mucosa associated with invagination of the small colon [9]. Parasitic burden has been identified as a major predisposing factor for the development of rectal prolapse [5]. The increasing prevalence of anthelmintic resistance in equine gastrointestinal parasites has been reported worldwide, particularly in cyathostomins and *Parascaris* spp., with occasional reports of reduced ivermectin efficacy [10]. Although the jenny was routinely treated with ivermectin plus praziquantel, parasitism cannot be completely excluded because fecal egg counts were not performed.

Rectal prolapse in donkeys has been associated with conditions that induce tenesmus or increase intra‐abdominal pressure, including cystic calculi, severe cough/bronchopneumonia, and dystocia [11]. Gastrointestinal disorders causing persistent straining, including enterocolitis, severe diarrhea, or proctitis, may also predispose equids to rectal prolapse [12]. Similarly, heavy gastrointestinal parasitism has been described as a contributing factor due to intestinal irritation and tenesmus [13, 14]. Additional factors related to the integrity and functionality of pelvic support tissues may also predispose animals to vaginal and rectal prolapse, including constipation, elevated estrogen levels during late pregnancy, exposure to estrogenic feeds, or ingestion of estrogenic fungal toxins [15].

Persistent anestrus observed in this case was likely multifactorial, potentially resulting from hormonal imbalances caused by physical stress and repeated prolapse episodes, as well as possible nutritional deficiencies that may impair reproductive function and tissue integrity. To the authors′ knowledge, this is the first reported case of postpartum vaginal and rectal prolapse in a jennet. This case highlights the potential for recurrence and complications associated with late‐pregnancy prolapses, the importance of timely intervention, and the possible long‐term reproductive consequences in affected jennets. Among the potential limitations of this report, the diagnosis was primarily based on clinical examination without additional laboratory or imaging diagnostics, which reflects the field conditions under which the case was managed. Measurement of the combined thickness of the uterus and placenta (CTUP) during late gestation was not assessed. Nevertheless, this report provides a detailed clinical description of a rare condition in donkeys and contributes valuable information regarding the presentation and management of concurrent vaginal and rectal prolapse in this species.

## 4. Conclusion

Vaginal prolapse in livestock is primarily associated with late‐stage pregnancy, hormonal fluctuation, and advanced age. Although the present case demonstrates that vaginal prolapse can occur during late gestation, further research is needed to fully elucidate the pathophysiological mechanisms contributing to both vaginal and rectal prolapse in postpartum female donkeys.

## Funding

The authors would like to thank CECAV and their support by the Projects UID/00772/2025 (doi:10.54499/UID/00772/2025) and LA/P/0059/2020 of the Portuguese Science and Technology Foundation (FCT).

## Consent

Informed consent for publication of this case report was obtained from the owner.

## Conflicts of Interest

The authors declare no conflicts of interest.

## Data Availability

The data used to support the findings of this study are included in this article.
